# Protease Activated Receptor 4 as a Novel Modulator of Regulatory T Cell Function

**DOI:** 10.3389/fimmu.2019.01311

**Published:** 2019-06-18

**Authors:** Qi Peng, Kulachelvy Ratnasothy, Dominic A. Boardman, Jacinta Jacob, Sim Lai Tung, Daniel McCluskey, Lesley A. Smyth, Robert I. Lechler, Anthony Dorling, Giovanna Lombardi

**Affiliations:** ^1^MRC Centre for Transplantation, Guy's Hospital, King's College London, London, United Kingdom; ^2^NIHR Biomedical Research Centre, Guy's Hospital, Guy's & St Thomas' NHS Foundation Trust, King's College London, London, United Kingdom; ^3^School of Health, Sport and Bioscience, University of East London, London, United Kingdom

**Keywords:** protease activated receptor 4 (PAR4), regulatory T cells (Tregs), immunoregulation, PI3K/Akt signaling pathway, phosphatase and tensin homolog on chromosome 10 (PTEN), forkhead box protein O1 (FoxO1)

## Abstract

Regulatory T cells (Tregs) are a subpopulation of T cells that maintain immunological tolerance. In inflammatory responses the function of Tregs is tightly controlled by several factors including signaling through innate receptors such as Toll like receptors and anaphylatoxin receptors allowing an effective immune response to be generated. Protease-activated receptors (PARs) are another family of innate receptors expressed on multiple cell types and involved in the pathogenesis of autoimmune disorders. Whether proteases are able to directly modulate Treg function is unknown. Here, we show using two complimentary approaches that signaling through PAR-4 influences the expression of CD25, CD62L, and CD73, the suppressive capacity, and the stability of Tregs*, via* phosphorylation of FoxO1 and negative regulation of PTEN and FoxP3. Taken together, our results demonstrate an important role of PAR4 in tuning the function of Tregs and open the possibility of targeting PAR4 to modulate immune responses.

## Introduction

Accumulated evidence suggests that both innate and adaptive immunity are involved in regulating inflammatory responses in diverse diseases including infection, autoimmunity and transplant rejection though precise mechanisms are still unclear (1). Tregs are immunosuppressive cells that maintain tolerance to self and immune balance (2, 3), as well as promoting tolerance toward alloantigens in a transplant setting (4, 5). Thymus-derived Tregs are characterized by a constitutively high expression of CD25 and the master transcription factor FoxP3. Mice deficient in FoxP3 exhibit systemic autoimmune disease and the Tregs have an impaired suppressive capacity *in vitro*, highlighting the importance of FoxP3^+^ Tregs *in vivo* (6–8).

The function of Tregs is tightly controlled *in vivo* to ensure that self-tolerance is maintained whilst protective immune responses can be elicited. Various molecular signals are capable of manipulating the function of Tregs including signaling through innate receptors such as Toll like receptors (TLRs) (9, 10) and anaphylatoxin receptors (C3aR/C5aR) (11, 12). Signaling through these receptors downregulates FoxP3 expression which causes Tregs to adopt an effector T cell function (13, 14), ultimately causing autoimmunity (15, 16), or transplant rejection (17–19). The precise mechanisms behind how these signaling pathways alter the function of Tregs are not fully understood, although recent studies have suggested PTEN is important. In Tregs, PTEN dephosphorylates Akt which enhances the activity of the transcription factor FoxO1, resulting in increased FoxP3 expression (20, 21).

Protease-activated receptors (PARs) are a family of innate G-protein-coupled receptors which comprises four members; PAR1, 2, 3, and 4 (22–25). PARs are expressed on a variety of cell types (26) and their expression is dramatically increased during infection, trauma and tissue necrosis (27–29). PARs are activated by serine proteases which cleave an extracellular region of the receptor, exposing an “internal ligand” which facilitates signal transduction. The serine protease thrombin, an important enzyme involved in hemostasis and thrombosis can activate PARs 1, 3, and 4 but the efficiency with which this is achieved differs between PARs. Importantly, higher concentrations of thrombin are required to activate PAR4 compared to PAR1 (25, 30).

Thrombin is an important modulator of inflammation. *In vivo* administration of thrombin has been shown to induce systemic autoimmunity (31) whilst inhibition of thrombin can ameliorate collagen induced arthritis (32), suggesting that serine proteases act as danger signals. Whilst some of the observed impact of thrombin is mediated via thrombosis, there are important lines of evidence to suggest that signaling through PAR is a predominant mechanism. Specifically, *in vivo* PAR1 activation causes mice to develop inflammatory bowel disease (IBD) whilst PAR1 inactivation or PAR1 deficiencies reduce the severity of IBD (33). Our previous work has highlighted the critical role that thrombin, acting predominantly through PAR1, plays in various models of acute and chronic vascular inflammation, being required for generation of local chemokine gradients and recruitment of inflammatory leukocytes (34, 35) and for production of growth factors involved in hyperplastic vascular disease (36). Furthermore, *in vivo* activation of PAR4 leads to inflammation and exaggerates ischemia reperfusion injury (37, 38). The immunoregulatory effects of PAR1 and PAR4 have also been demonstrated in experiments where the action of serine proteases was blocked by the administration of protease inhibitors α1-antitrypsin (AAT) or anti-thrombin III (ATIII). Treatment with AAT prevented allogeneic islet transplant rejection and the development of experimental autoimmune encephalomyelitis (EAE) and correlated with an increase in the number of FoxP3^+^ cells *in vivo* (39, 40). Similarly, ATIII administration prevented hyperacute lung rejection and induced indefinite survival of heart allografts, but only at a high dose (500 U/Kg) (41, 42). Finally, we have previously demonstrated the immunoregulatory influence of PAR2 signaling on antigen presenting cells and CD4 T cells in mouse models of inflammation (43). These studies suggest that modulation of serine protease activity or PAR signaling promotes immunoregulation via downstream signaling pathways on multiple cell types.

In this study, we assessed whether the presence or absence of signaling through PAR4 impacted Treg function and influenced immunoregulation. We show that Tregs from mice deficient in PAR4 or WT Tregs following treatment with a PAR4 antagonist, express higher levels of key regulatory molecules and exhibit an enhanced suppressive capacity *in vitro* and *in vivo*. Furthermore, we demonstrate that activation of PAR4 on murine Tregs inhibits their function via Akt dependent phosphorylation of the transcription factor FoxO1 which, in turn, negatively regulates PTEN and FoxP3. Altogether, the data presented here demonstrate that PAR4 signaling inhibits the function of Tregs and suggest that targeting PAR4 on Tregs can be a novel therapeutic strategy.

## Materials and Methods

### Mice

WT C57BL/6 (WT), BALB/c and transgenic CD45.2^+^ C57BL/6 mice were purchased from Charles River. Transgenic CD45.1^+^ C57BL/6, CD45.2^+^*Rag2*^−/−^ and PAR4^−/−^ mice were maintained under sterile conditions (Biological Services Unit, New Hunt's House, King's College London). Procedures were carried out in accordance with all legal, ethical and institutional requirements (PPL70/7302).

### Treg Isolation and Culture

CD4^+^CD25^+^ T cells were enriched from the spleen and LNs of WT C57BL/6 or PAR4ko mice either by cell sorting, or using the Dynabeads Flowcomp murine CD4^+^CD25^+^ Treg cells kit (44), as per manufacturer's protocols (ThermoFisher Scientific, Paisley, Renfrewshire, UK). The purity of CD4^+^CD25^+^ cells by both methods were over 95% (Supplementary Figures 2A,B). Enriched Tregs were stimulated using either plate-bound anti-CD3 (2 μg/mL, BD Biosciences, Franklin Lakes, NJ, USA) and anti-CD28 (4 μg/mL, R&D systems, Abingdon, Oxfordshire, UK), for short-term cultures (3–6 days) or in suppression assay *in vitro* (described below). All cells were cultured in complete media consisting of RPMI-1640 supplemented with 10% fetal calf serum, 100 U/mL penicillin, 100 μg/mL streptomycin, 2 mM L-glutamine, 10 mM HEPES and 50 μM β2-mercaptoethanol (all from ThermoFisher Scientific) and Treg cultures were supplemented with 10 U/mL recombinant human IL-2 (Proleukin-Novartis, Camberley, Surrey, UK).

### Flow Cytometry and Cell Sorting

Cells were stained with fluorescently-conjugated antibodies specific for CD19 (clone: 1D3), CD4 (clone: GK1.5), CD8 (clone: 53-6.7), CD25 (clone: PC61.5), FoxP3 (clone: FJK-16s), CTLA4 (clone: UC10-4B9), CD103 (clone: 2E7; all from ThermoFisher Scientific); CD3 (clone: 145-2C), CD62L (clone: MEL-14), CD73 (clone: TY/11.8), CD45.1 (clone: A20), CD45.2 (clone: 104; all from Biolegend, London, UK); Neuropilin-1 (R&D, Minneapolis, MN, USA); pSTAT5 (Cell Signaling Technology, Danvers, MA, USA) and PAR4 (Bioss Antibodies, Woburn, MA, USA). Dead cells were excluded using LIVE/DEAD Fixable Near-IR Dead Cell Stain Kit (ThermoFisher Scientific). Intracellular staining of FoxP3 and CTLA4 was performed using the FoxP3/Transcription Factor Staining Buffer kit (eBioscience, Santa Clara, CA, USA). Cells were acquired and sorted using LSRFortessa II and FACSAria II, respectively. Data were analyzed using FlowJo 10 software (Tree Star, Ashland, OR, USA). For detection of PAR4, Tregs were first stained for CD4 and CD25, fixed/permeabilized and stained for PAR4 and analyzed by flow cytometry. For detection of phosphorylated STAT5, Tregs were starved for 2 h at 37°C before being cultured in the presence of 20 U/mL IL-2 for 30 min. Cultured cells were harvested and fixed/permeabilized for 10 min before being incubated with an unconjugated pSTAT5 antibody followed by detection antibody (anti-rabbit IgG-AF-488, ThermoFisher Scientific) and analyzed by flow cytometry.

### Detection of PAR4 Expression

Total RNA was isolated from Tregs using TRIzol® Reagent, as per manufacturer's protocols (ThermoFisher Scientific), and cDNA was subsequently generated using the GoScript Reverse Transcription system (Promega UK, Southampton). PCR reaction was performed using 2 μL cDNA (reflecting 0.2 μg total RNA), 12.5 pmol of a forward (FWD) and reverse (REV) primer and GoTaq^®^ Master Mixes (Promega) with the following the cycling condition: denaturation at 95°C (20 s), annealing at 60°C (45 s) and extension at 72°C (20 s); 40 cycles. PAR4 expression was examined with FWD 5′-GTACCAGGGGAAGCCATGAAG-3′ and REV 5-TCATGAGCAGAATGGTGGATGG-3′ primers (MGI: 1298207) and 18S as a housekeeping gene with FWD 5′-AGAAACGGCTACCACATCCA-3′ and 5′CCCTCCAATGGATCCTCGTT-3′ primers (GeneBank: X56974). Amplified PCR products (195 bp for PAR4 and 158bp for 18S) were visualized on 1.5% agarose gels.

### *In vitro* Suppression Assay

The suppressive activity of Tregs was determined by measuring their ability to inhibit PAR4ko CD4^+^CD25^−^ responder T cells (Tresps) proliferation upon co-culture with PAR4ko antigen presenting cells (APCs). Tresps were isolated from the spleen and LNs of PAR4^−/−^ mice using the murine CD4^+^CD25^+^ Treg cells kit (ThermoFisher Scientific) as per manufacturer's protocols. Similarly, APCs were isolated from the spleen and LNs of PAR4^−/−^ C57BL/6 mice using a negative selection protocol established in the lab (45) whereby T cells were labeled using anti-CD4 and anti-CD8 antibodies (YTS-191 and YTS-169, respectively) and removed using anti-rat IgG-coated beads and a magnet.

Suppression assays were set up by co-culturing 0.5 ×10^5^ WT Tresps with 1 ×10^5^ WT APCs in the presence of 1 μg/ml anti-CD3_ε_ antibody (ThermoFisher Scientific). WT or PAR4^−/−^ Tregs were added to co-cultures such that desired Treg:Tresp ratios were obtained.

In some assays where PAR4 on Tregs was blocked or stimulated, WT Tregs were used and co-cultured with KO Tresps at a 1:2 Treg:Tresp ratio in the presence of KO APCs. Mouse PAR4 antagonist (*trans*-Cinnamoyl-Tyr-Pro-Gly-Lys-Phe-NH_2_, tcY-NH_2_) or agonist (Ala-Tyr-Pro-Gly-Lys-Phe-NH_2_, AY-NH_2_; Bio-Techne, **Minneapolis**, MN, USA) were used at a concentration of 50 μM and human α-thrombin (HTI; Essex Junction, VT, USA) was used at concentrations from 10 to 200 nM. Thrombin inhibitor hirudin (200 nM; Sigma-Aldrich, MO, USA) or protease inhibitor cocktail (0.1%; Sigma-Aldrich, MO, USA) was also used.

Co-cultures were incubated at 37°C (5% v/v CO_2_) for 72 h after which Tresp proliferation was measured. This was accomplished either by CFSE dye dilution, where Tresps were labeled with 0.5 μM CFSE prior to co-culture, or by ^3^H-thymidine incorporation, where 1 μCi/well ^3^H-thymidine (Perkin Elmer UK, Beaconsfield) was added for the last 18 h of culture.

### Cytokine Analysis

IL-2, IFNγ, IL-10, and TNFα production was measured in culture supernatants using a cytometric bead array (CBA, BD, Oxford, Oxfordshire, UK), as per manufacturer's protocols.

### Western Blot

Tregs were isolated and incubated in complete RPMI medium for 2 h at 37°C prior to assays being performed. WT cells were then stimulated with 50 μM mouse PAR4 agonist for 15 min at 37°C. Alternatively, WT and PAR4ko Tregs were stimulated with plate-bound anti-CD3 and anti-CD28 antibodies for various time points at 37°C. Cell lysates were prepared in RIPA Lysis and Extraction buffer (ThermoFisher Scientific) with protease inhibitor cocktail (Merck) and proteins were separated on pre-casted 4–12% mini gels (ThermoFisher Scientific) before being transferred to immunoblots. Blots were blocked in 5% BSA and labeled with primary antibodies specific for p-Akt (Ser473), Akt, p-FoxO1 (Thr24), PTEN, and β-actin followed by secondary HRP-conjugated antibodies (Cell Signaling Technology), according to manufacturer's recommendations. Results were quantified by densitometry.

### Skin Transplantation

Rag2^−/−^ mice were reconstituted with 4 ×10^5^ CD45.1^+^CD4^+^CD25^−^ Tresps ± 2 ×10^5^ CD45.2^+^CD4^+^CD25^+^ Tregs by intravenous injection 1 day prior to transplantation. Skin transplantation was then performed as previously described (44). Briefly, BALB/c tail skin grafts were transplanted onto the Rag2^−/−^ mice and protected with a plaster cast for 7 days. Grafts were then monitored every 2–3 days for signs of rejection which was defined as >80% necrosis, assessed by visual inspection. Histological analyses were performed by H&E staining on grafts which were rejected or harvested at day 65 or day 135 post-transplant using Olympus BX51 microscope. The proportion of CD45.1^+^ and CD45.2^+^ FoxP3^+^ cells in the draining LNs was analyzed by flow cytometry at day 115.

### Statistics Analysis

Data are expressed as mean ± SEM and statistical significance was determined using an unpaired two-tailed Student's *t-*test or a two-way ANOVA as stated. In transplant experiments, statistical differences in the survival time of grafts was determined using a log-rank test.

## Results

### In the Absence of PAR4 Signaling Tregs Express Higher Levels of CD25, CD73, and CD62L Molecules and Are More Stable

**Figure 1:**
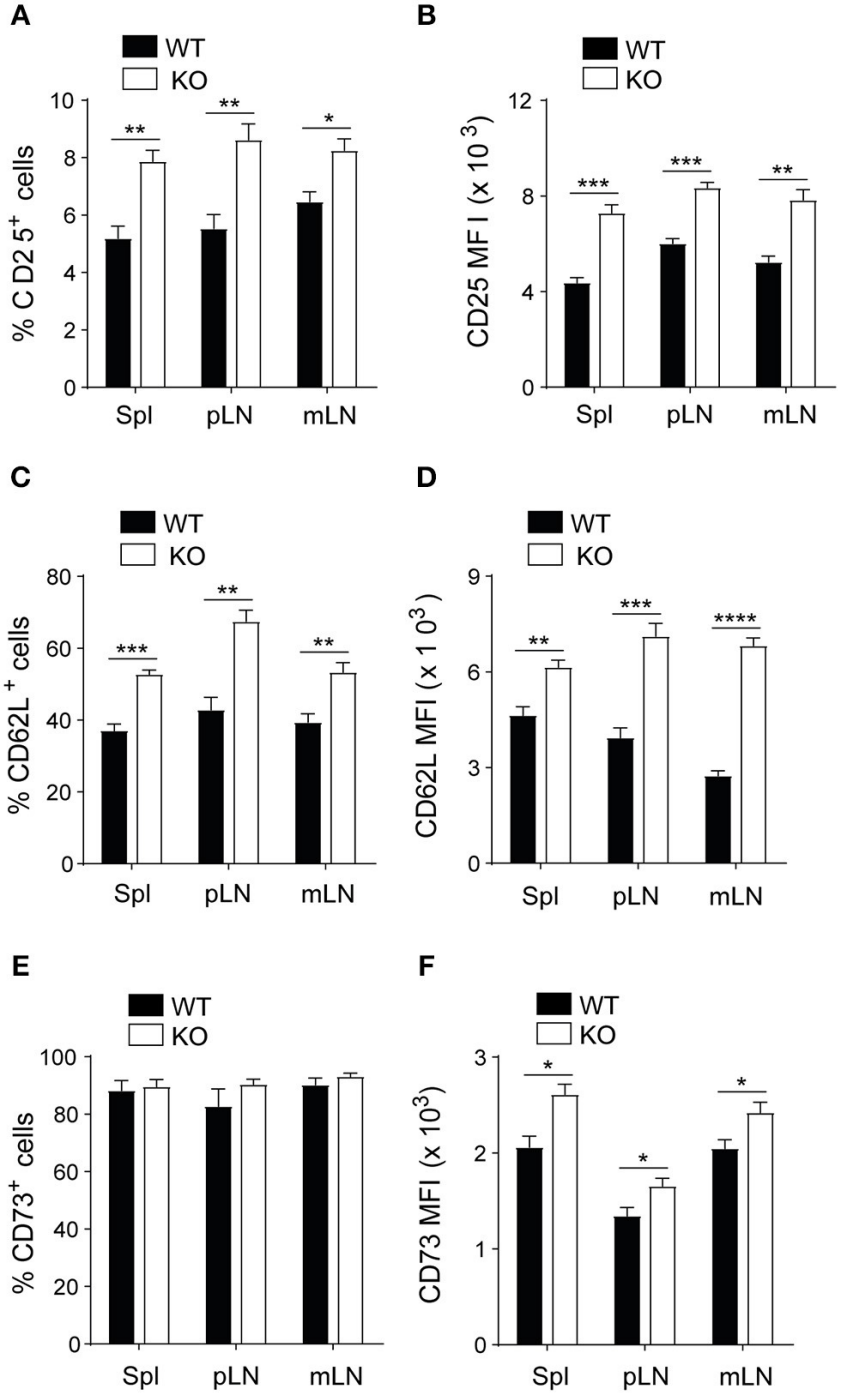
Tregs from PAR4ko mice express higher levels of CD25, CD62L, and CD73 molecules. Cells obtained from homogenized the spleen, peripheral lymph nodes (pLN) and mesenteric lymph nodes (mLN) of C57BL/6 WT (filled bars) and PAR4ko (open bars) mice were analyzed using flow cytometry. The percentage of CD25^+^ cells in the CD4^+^ T cells **(A)** and within the CD4^+^CD25^+^ T cells the percentages of CD62L and CD73 **(C,E)** are shown. The MFI levels for the same populations of these cells in **(A,C,E)** are shown in **(B,D,F)**. Graphs represent mean ± SEM from six mice per group. Data were analyzed by unpaired two-tailed *t*-test. ^*^p < 0.05, ^**^p < 0.005, ^***^p < 0.0005, and ^****^p < 0.0001 when WT and PAR4ko Tregs were compared.

To investigate whether the expression of PAR4 influences Treg phenotype, function and stability, these cells were studied in mice lacking PAR4 (PAR4ko) and compared with the same population in wild-type (WT) mice. The percentage of CD4^+^CD25^+^ T cells were analyzed in the spleen (Spln) and peripheral lymph nodes (LNs) by flow cytometry (gating strategy shown in Supplementary Figure 1A). PAR4ko mice had significantly higher percentages of CD4^+^CD25^+^ T cells (*p* < 0.05 and *p* < 0.005, respectively; Figure 1A) with increased levels of CD25 expression (MFI) compared to WT Tregs (*p* < 0.005 and *p* < 0.0005, respectively; Figure 1B). Next, we extended our analysis to other important markers knowing to influence Treg function. By gating on the CD4^+^CD25^+^ T cells, we observed that the percentages of Tregs expressing CD62L^+^ (*p* < 0.005 and *p* < 0.0005, respectively; Figure 1C and Supplementary Figure 1B) but not CD73^+^ (Figure 1E and Supplementary Figure 1C) were significantly higher in PAR4ko compared to WT mice. However, when the expression levels of CD62L and CD73 were analyzed on CD4^+^CD25^+^ T cells, they were both significantly higher in PAR4ko compared to WT animals (*p* < 0.05, *p* < 0.005, *p* < 0.0005, and *p* < 0.0001, respectively; Figures 1D,F). In contrast, there were no significant differences in FoxP3, CTLA-4, neuropilin^+^ and CD103^+^ expression on CD4^+^CD25^+^ T cells between WT and PAR4ko mice (Supplementary Figures 1D–G). Overall, these observations suggest that Tregs derived from mice lacking PAR4 have an increased percentage and expression of some of the molecules known to relate to the function of Tregs.

**Figure 2:**
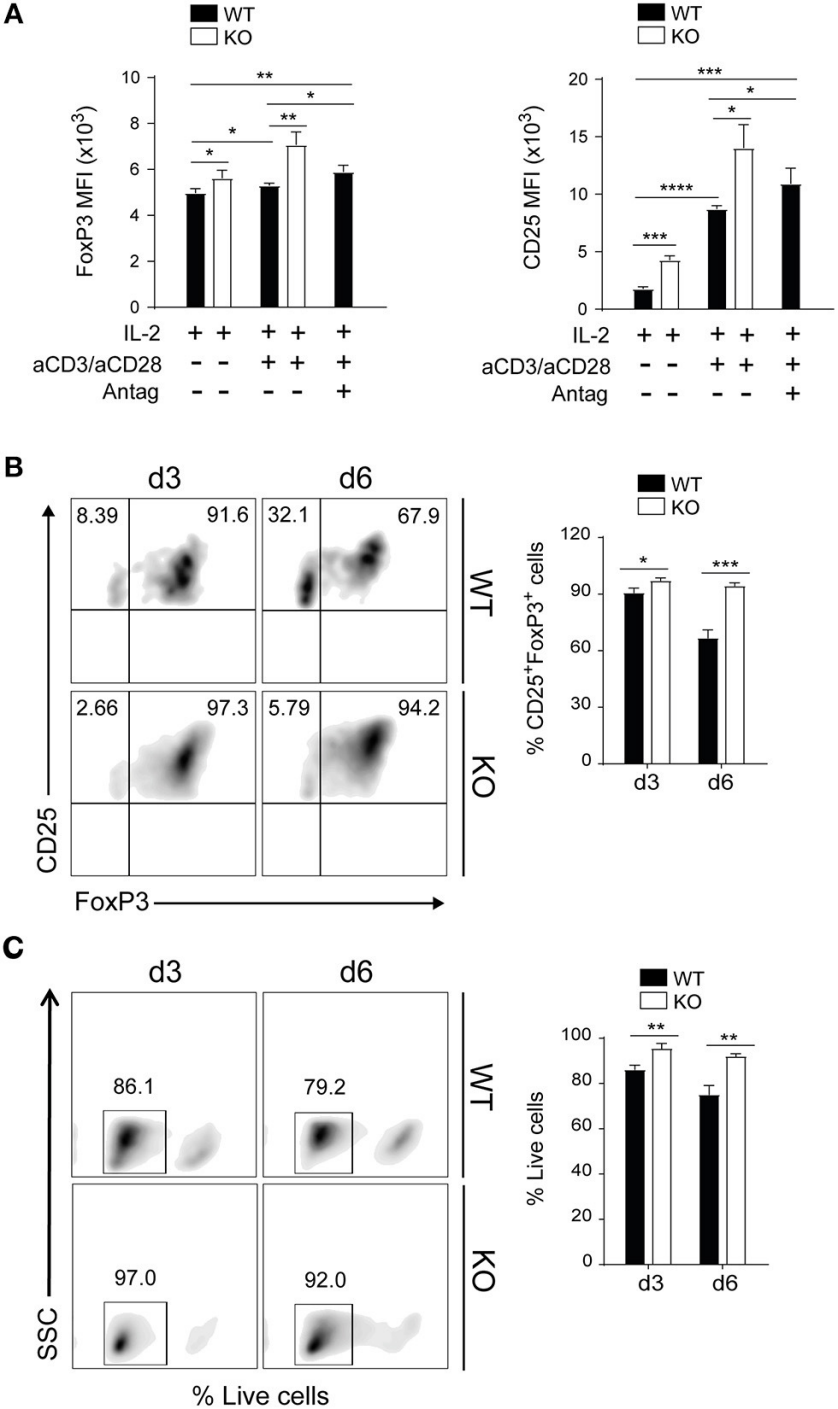
PAR4 signaling regulates stability and survival of Foxp3^+^ Tregs *in vitro*. CD4^+^CD25^+^ Tregs were freshly isolated from spleen and pLNs of WT (filled bars) and PAR4ko (open bars) mice. The Tregs were activated with anti-CD3/anti-CD28 in the presence of IL-2 for 3 days and in some cases PAR4 antagonist (Antag) was added to WT Tregs. The MFI levels of FoxP3 and CD25 expression were analyzed by flow cytometry **(A)**. Flow cytometry assessment of FoxP3 and CD25 expression on Tregs activated by anti-CD3/anti-CD28 in presence of IL-2 for 3 and 6 days **(B)**. Flow cytometry assessment of live Tregs activated by anti-CD3/anti-CD28 in presence of IL-2 for 3 and 6 days **(C)**. The cells were stained with live/dead near-IR dead cell stain kit. Graphs show mean ± SEM from four experiments. Data were analyzed by unpaired two-way *t*-test. ^*^p < 0.05, ^**^p < 0.005, ^***^p < 0.0005, and ^****^p < 0.0001 in comparison between WT and PAR4ko Tregs.

To further dissect the role of PAR4 in Tregs, these cells were purified from the spleen LNs of WT and PAR4ko mice. The percentage of purity achieved was between 95 and 98% and all the T cells that were CD4^+^ and CD25^+^ were also FoxP3^+^ (Supplementary Figures 2A,B). The lack of expression of PAR4 on highly purified Tregs from PAR4ko mice was confirmed by PCR and flow cytometry (Supplementary Figures 2C,D) as well as on total CD4^+^ T cells (data not shown). Purified Tregs (CD4^+^CD25^+^ T cells) were then activated *in vitro* with IL-2 alone or with a combination of plate-bound anti-CD3/anti-CD28 antibodies and IL-2. After 3 days of incubation, the levels of expression of FoxP3 and CD25 were significantly higher on purified Tregs derived from PAR4ko mice compared to WT Tregs (*p* < 0.05, *p* < 0.005, *p* < 0.0005, and *p* < 0.0001, respectively; Figure 2A). To confirm that the higher expression observed in PAR4ko Tregs was due specifically to the lack of PAR4 in Tregs, WT Tregs were activated with plate-bound anti-CD3/anti-CD28 antibodies and IL-2 but also in the presence of a PAR4 antagonist (tcY-NH2; Antag). As shown in Figure 2A the levels of FoxP3 and CD25 expression on the purified Tregs were increased in the presence of the PAR4 antagonist.

Next, the stability of FoxP3 expression on WT and PAR4ko Tregs was analyzed. After 3 and 6 days of activation with anti-CD3/CD28 and IL-2, the proportions of WT Tregs expressing FoxP3 was reduced from 90 ± 0.8% at 3 days to 66 ± 2.4% by day 6, whereas FoxP3 was maintained in PAR4ko (94 ± 0.9% at 3 days and 90 ± 1.4% at day 6) (*p* < 0.05 and *p* < 0.0005, respectively; Figure 2B). Overall, these results highlight the positive impact that the absence of PAR4 has on the phenotype and stability of Tregs.

Finally, the cell viability of WT and PAR4ko Tregs were analyzed using live/dead near-IR dead cell stain kit. After 3 and 6 days of activation with anti-CD3/CD28 and IL-2, PAR4ko Tregs had significantly less dead cells compared to WT group (*p* < 0.005, Figure 2C) at day 3 and 6 after activation, suggesting a role of PAR4 signaling in cell survival.

### Tregs in the Absence of PAR4 Have an Augmented Suppressive Capacity Compared to WT Tregs *in vitro*

**Figure 3:**
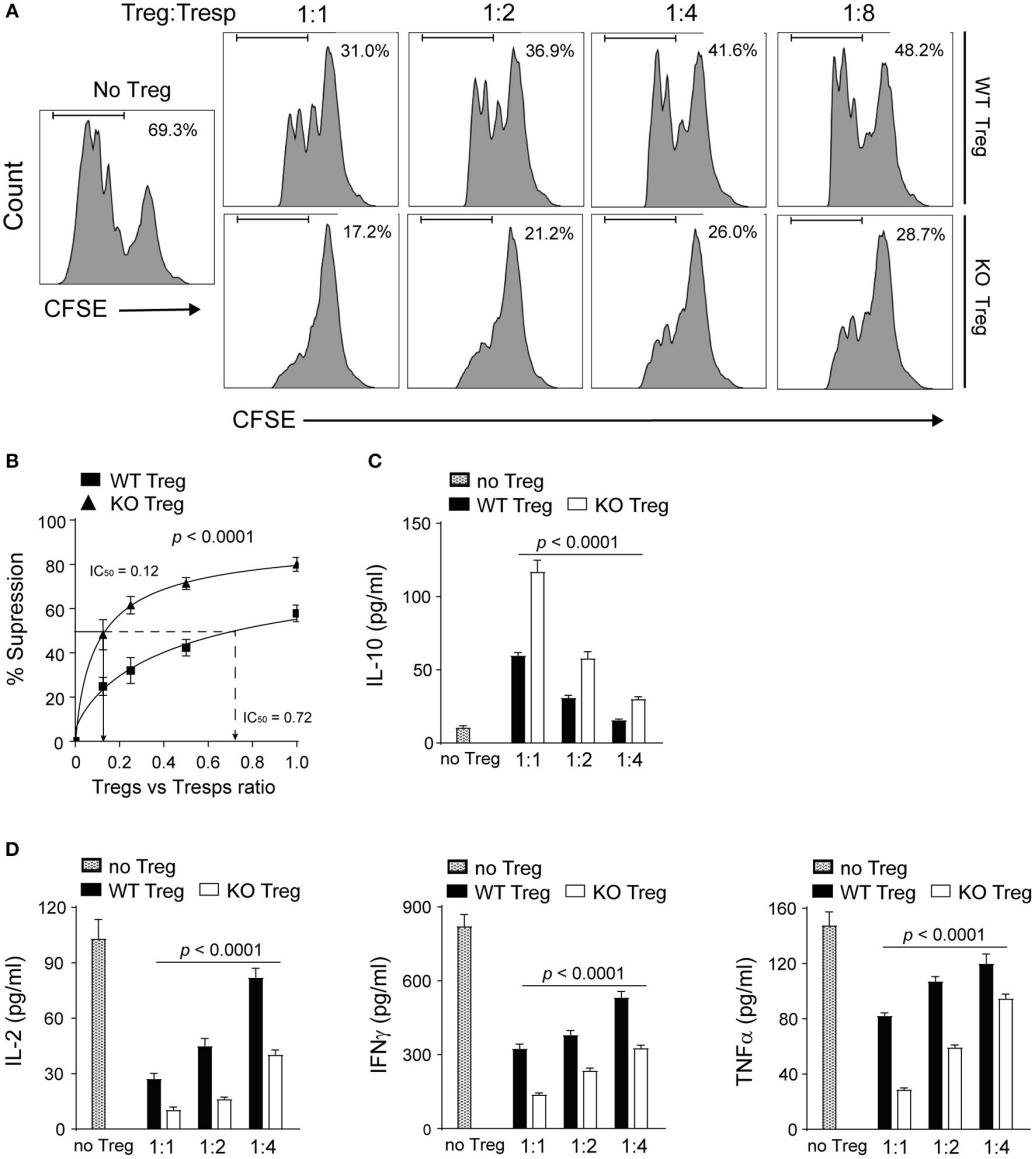
Absence of PAR4 leads to the enhanced suppressive capacity of Tregs. CD4^+^CD25^+^ Tregs from WT spleen and pLN were purified and co-cultured with PAR4ko Tresps and APCs in the presence of anti-CD3 for 72 h. Representative histograms showing the CFSE dilutions of Tresps cultured alone (No Tregs) or with different numbers of WT or PAR4ko Tregs **(A)**. Pooled data showing the percentages of suppression of WT and PAR4ko Tregs [*p* < 0.0001; **(B)**]. IC50 values representing the Treg:Tresps ratio required to achieve 50% suppression are shown for the PAR4ko (triangles) and WT (squares) Tregs. Graphs show mean ± SEM from six experiments. Data were analyzed by two-way ANOVA to compare between these two Treg groups. CBA analysis of cytokines IL-10 [*p* < 0.0001; **(C)**] and IL-2, IFNγ, and TNFα [*p* < 0.0001; **(D)**] in the supernatants obtained from six suppression assays. Graphs show mean ± SEM from four experiments. Data were analyzed by two-way ANOVA to compare between these two Treg groups with a sequential dilution.

Next we investigated the impact of PAR4 on the suppressive capacity of Tregs. Highly pure Tregs isolated from the spleen and pLNs of WT and PAR4ko mice were co-cultured with CFSE-labeled responder CD4^+^CD25^−^ T cells (Tresps) and splenic antigen-presenting cells (APCs) isolated from PAR4ko mice in the presence of anti-CD3 antibody (45). Tregs from PAR4ko mice were significantly more efficient at suppressing the proliferation of Tresps compared to WT Tregs (*p* < 0.0001; Figures 3A,B). To quantify the suppressive potency of Tregs, the Treg:Tresp ratio required to suppress 50% of the Tresps proliferation (IC_50_) was calculated (46). PAR4ko Tregs had an IC_50_ value of 0.12 compared to 0.72 for WT Tregs (Figure 3B), suggesting that PAR4ko Tregs were 6-fold more efficient at suppressing Tresps proliferation compared to WT Tregs. The same results were obtained when the inhibition of Tresps proliferation was measured using thymidine incorporation (Supplementary Figures 3A,B).

Furthermore, the analysis of the cytokines revealed that the immunosuppressive cytokine IL-10 was present in significantly higher quantities in the supernatants of the Treg:Tresp co-cultures when the Tregs were derived from PAR4ko compared to WT Tregs (*p* < 0.0001; Figure 3C) whereas the opposite was observed for IL-2, IFNγ and TNFα (*p* < 0.0001; Figure 3D), further supporting the superior immunomodulatory function of PAR4ko Tregs compared to WT Tregs *in vitro*.

**Figure 4:**
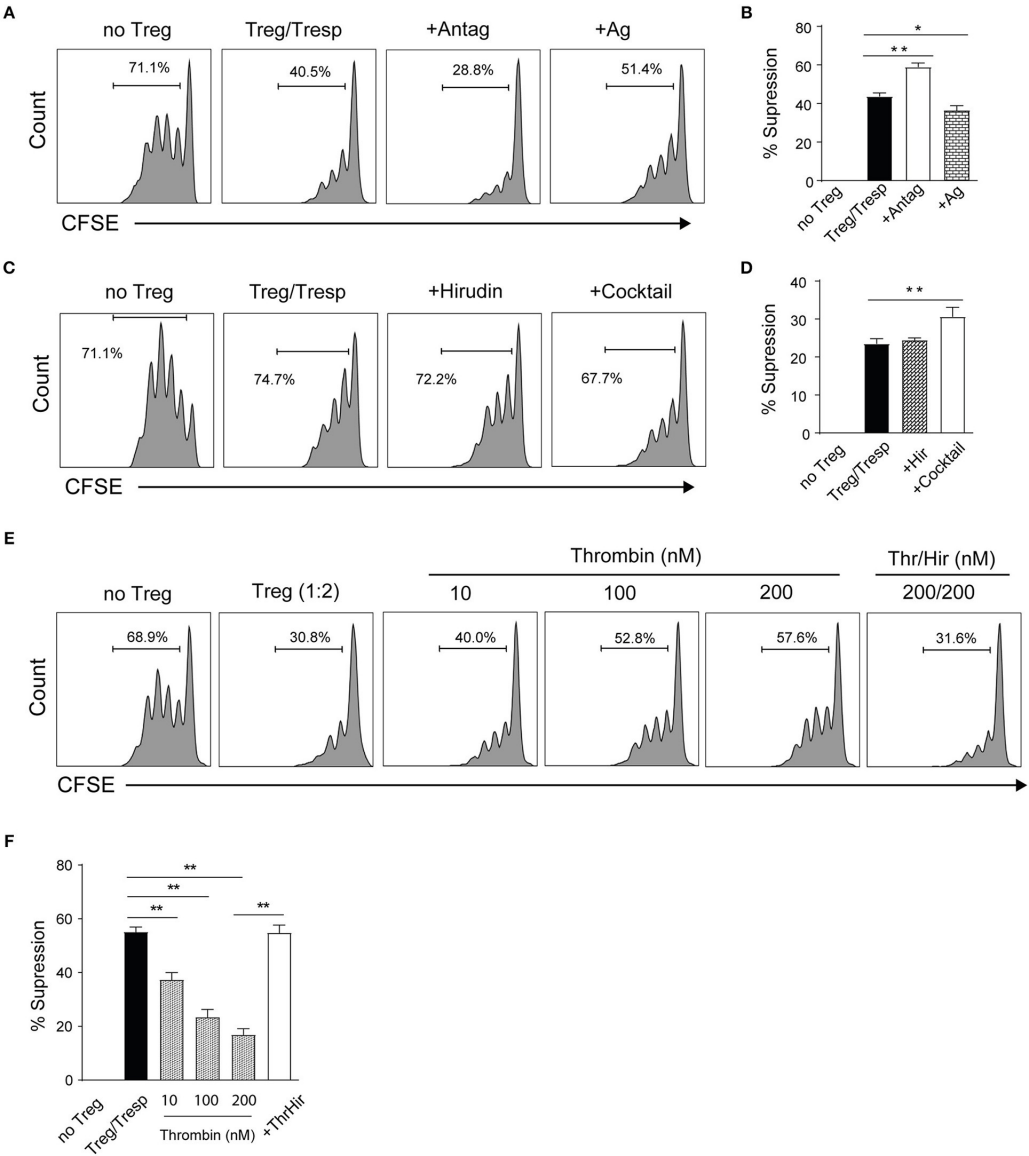
PAR4 signaling modulates the suppressive capacity of Tregs. CD4^+^CD25^+^ Tregs from WT spleen and pLN were freshly isolated. Representative histograms **(A)** and pooled data **(B)** showing *in vitro* suppression of CFSE-labeled PAR4ko Tresps by WT Tregs (filled bar; in the ratio of 2 to 1) in the absence or presence of a PAR4 antagonist (Antag; open bar) or agonist (Ag; dotted bar) in 72 h suppressive assays co-cultured with PAR4ko APCs and addition of anti-CD3. Graphs show mean ± SEM from four experiments. Data were analyzed by unpaired two-way *t*-test. ^*^p < 0.05 and ^**^p < 0.01 in comparison between the Treg groups without or with PAR4 antagonist or agonist, respectively. Representative histograms **(C)** and pooled data **(D)** showing *in vitro* suppression of CFSE-labeled PAR4ko Tresps by WT Tregs (filled bar; in the ratio of 2 to 1) in the absence or presence of thrombin inhibitor hirudin (Hir; dotted bar) or protease inhibitor cocktail (open bar) in 72 h suppressive assays. Graphs show mean ± SEM from four experiments. Data were analyzed by unpaired two-way *t*-test. ^**^p < 0.01 in comparison between the Treg groups without or with the cocktail, respectively. Representative histograms **(E)** and pooled data **(F)** showing *in vitro* suppression of CFSE-labeled PAR4ko Tresps by WT Tregs (in the ratio of 2 to 1) in the absence (filled bar) or presence of thrombin (Thr) in different concentrations without (dotted bars) or with Hir (open bars). Graphs show mean ± SEM from four experiments. Data were analyzed by unpaired two-way *t*-test. ^**^p < 0.01 in comparison between the WT Tregs without or with thrombin.

To further validate the influence of PAR4 on Treg function, the suppressive ability of WT Tregs was evaluated in the presence of the PAR4 antagonist (Antag) tcY-NH_2_ or agonist (Ag) AY-NH_2_ (30, 47). As predicted, the suppressive capacity of WT Tregs was significantly enhanced in the presence of the antagonist (*p* = 0.0013; Figures 4A,B). In contrast, activation through PAR4 by the PAR4 agonist decreased the suppressive ability of the WT Tregs (*p* = 0.0136; Figures 4A,B). To confirm the specificity of PAR4 antagonist, the suppression assays were set up with PAR4ko Tregs in the absence or in the presence of the antagonist. No significant effect on the suppressive capacity of PAR4ko Tregs was observed (Supplementary Figures 4A,B).

To investigate which component in the culture medium was binding to PAR4 and allowed signaling through this receptor *in vitro*, we explored first whether thrombin, a natural ligand for PAR4, was present in culture and was responsible for the differences in the suppressive ability observed in the presence or absence of a functional PAR4 on Tregs. The suppression assays were performed with WT Tregs in the presence of highly specific thrombin inhibitor hirudin (Hir, 200 nM) or protease inhibitor cocktail (known to inhibit serine and cysteine proteases as well as aminopeptidases; 0.1%). As shown in Figures 4C,D, the presence of the protease inhibitor cocktail but not hirudin could enhance the suppressive capacity of WT Tregs (*p* = 0.0066), suggesting that an unidentified protease other than thrombin was responsible for our observations. However, to confirm that thrombin has an effect on the suppressive capacity of WT Tregs, thrombin was titrated into the suppression assays as shown in Figures 4E,F. The suppressive ability of WT Tregs was reduced in a dose-dependent manner (*p* = 0.0074, *p* = 0.0051, and *p* = 0.0020, respectively) and this effect was completely abolished by the addition of hirudin (*p* = 0.0011). These results are consistent with the hypothesis that an endogenous protease is responsible for the baseline activation of PAR4 on WT Tregs.

### PAR4ko Tregs Have a Superior Suppressive Capacity Compared to WT Tregs *in vivo*

**Figure 5:**
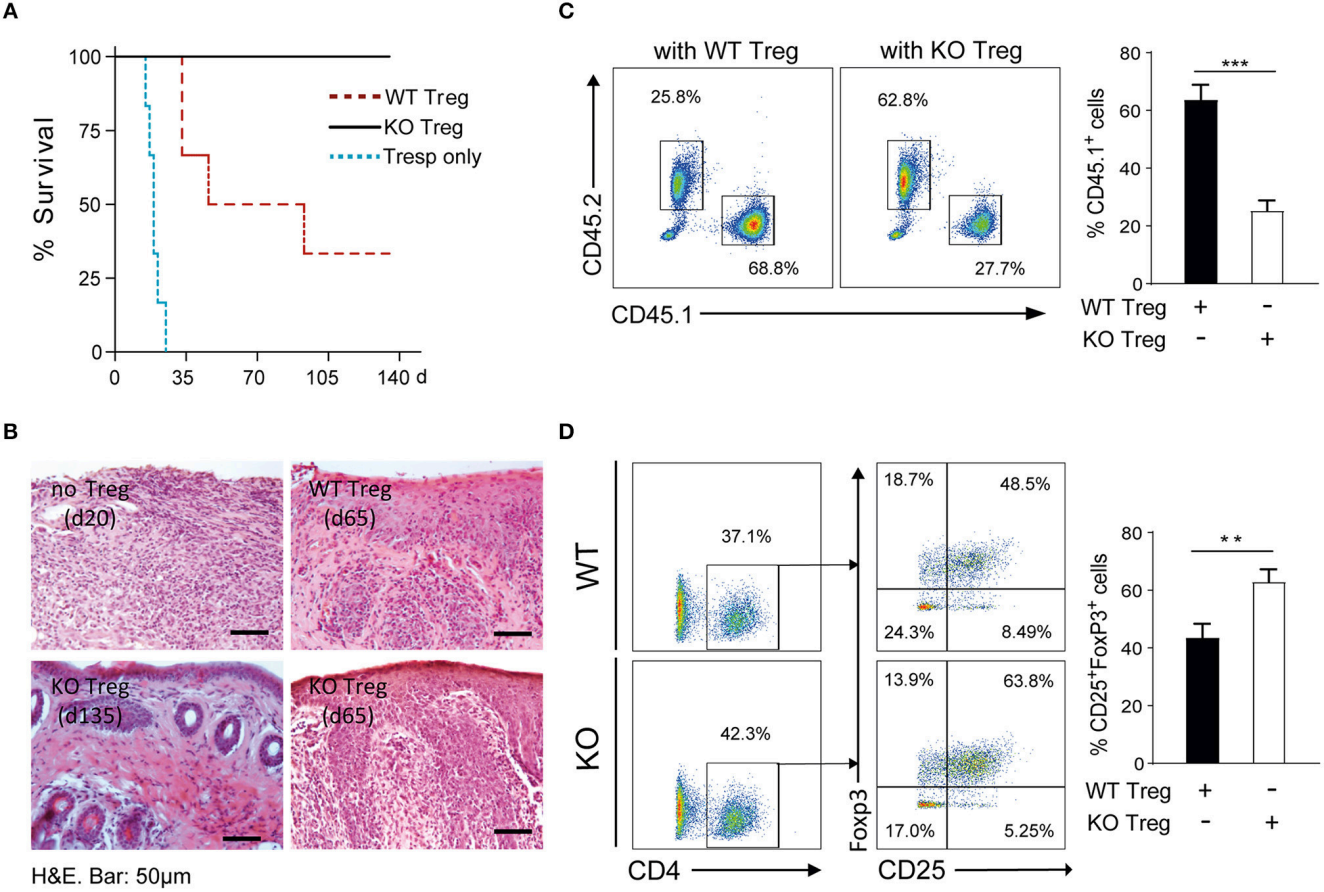
PAR4ko Tregs promote indefinite skin allograft survival *in vivo*. BALB/c skin graft survival was assessed in recipients Rag2^−/−^ mice in which CD45.1^+^ Tresps only (blue dotted line, *n* = 6) or at a ratio 2 to 1 with CD45.2^+^ WT (red dotted line, *n* = 6) or PAR4ko (black solid line, *n* = 6) Tregs were adoptively transferred. **(A)** Survival graph of skin allografts. Data were analyzed by Log-rank test. *p* = 0.0005, when Tresps with WT (or PAR4ko) Tregs were compared to Tresps only groups; *p* = 0.0179, comparison between WT and PAR4ko Treg groups. **(B)** Representative H&E staining of skin grafts from recipient mice receiving Tresps only (day 20) or Tresps with WT or PAR4ko Tregs (d65), or from Tresps with PAR4ko Tregs (d135). Bar, 50 μm. Flow cytometry analysis of CD45.1^+^ and CD45.2^+^ population **(C)** and the CD25^+^FoxP3^+^ cells in the CD4^+^ gated population of CD45.2^+^ cells **(D)** in the draining lymph nodes of the recipients receiving WT or PAR4ko Treg cells sacrificed at d115. Graphs show mean ± SEM from four mice per group. Data were analyzed by unpaired two-way *t*-test. ^**^p < 0.01 ^***^p < 0.001 when WT and PAR4ko Treg groups were compared.

Given our initial *in vitro* observations of the consequences of PAR4 expression on the regulatory ability of Tregs, the function of Tregs was assessed *in vivo* using an adoptive transfer model combined with a fully MHC-mismatched skin transplant model. Rag2^−/−^ mice were reconstituted with CD45.1^+^ Tresps with or without CD45.2^+^ WT or PAR4ko Tregs (2:1 Tresp:Treg ratio) and then transplanted with BALB/c tail skin grafts after 24 h (44). Skin grafts on mice that received Tresps only were rejected with a mean survival time (MST) of 19 days (blue dotted line, Figure 5A). Compared to the Tresps only group, skin graft survival was significantly prolonged (MST = 70 days; *p* = 0.0005) when recipient mice received WT Tregs (red dotted line, Figure 5A), while indefinite survival was achieved when PAR4ko Tregs were administered (MST > 135 days, black solid line, Figure 5A; *p* = 0.0005).

The morphology and cellular infiltration of the skin grafts was assessed histologically with H&E staining. Skin grafts from mice that received Tresps alone showed a dramatic loss of the skin architecture and an extensive cell infiltration at 20 days post-transplantation (Top left, Figure 5B). Conversely, grafts of mice that received Tresps with WT or PAR4ko Tregs displayed a healthier morphology and fewer infiltrated cells in PAR4ko Treg group at 65 days post-transplantation (Top right and bottom right, respectively, Figure 5B). Importantly, the skin grafts from mice that received PAR4ko Tregs the skin architecture was nearly normal and very few infiltrating cells at 135 days post-transplantation (Bottom left, Figure 5B). Furthermore, the analysis of the draining LNs 115 days after transplantation revealed a fewer number of CD45.1^+^ CD4^+^CD25^−^ (*p* = 0.0006; Figure 5C) and a higher number of CD45.2^+^ CD4^+^CD25^+^FoxP3^+^ cells (*p* = 0.0071; Figure 5D) in recipient mice that have received PAR4ko Tregs compared to mice that received WT Tregs.

Altogether, these findings suggest that Tregs expressing PAR4 are less potent in promoting skin transplant survival, highlighting a functional role for this receptor in Tregs *in vivo*.

### PAR4 Signaling Negatively Regulates the Activity of FoxO1 and STAT5 via Akt

Having shown that in the absence of PAR4, Tregs had higher expression of functional markers, increased stability and augmented suppressive abilities both *in vitro* and *in vivo*, the mechanisms by which PAR4 regulates Treg function were investigated. Previous studies have indicated that PAR4 engagement activates downstream PI3K/Akt signaling cascades in platelets (47). In Tregs it has been reported that increased PI3K signaling and downstream Akt phosphorylation (48) inhibited FoxP3 expression through the downregulation of PTEN, a potent negative regulator of PI3K (49, 50). Given this we hypothesized that PAR4 signaling might control FoxP3 expression by altering the Akt and PTEN pathways.

**Figure 6:**
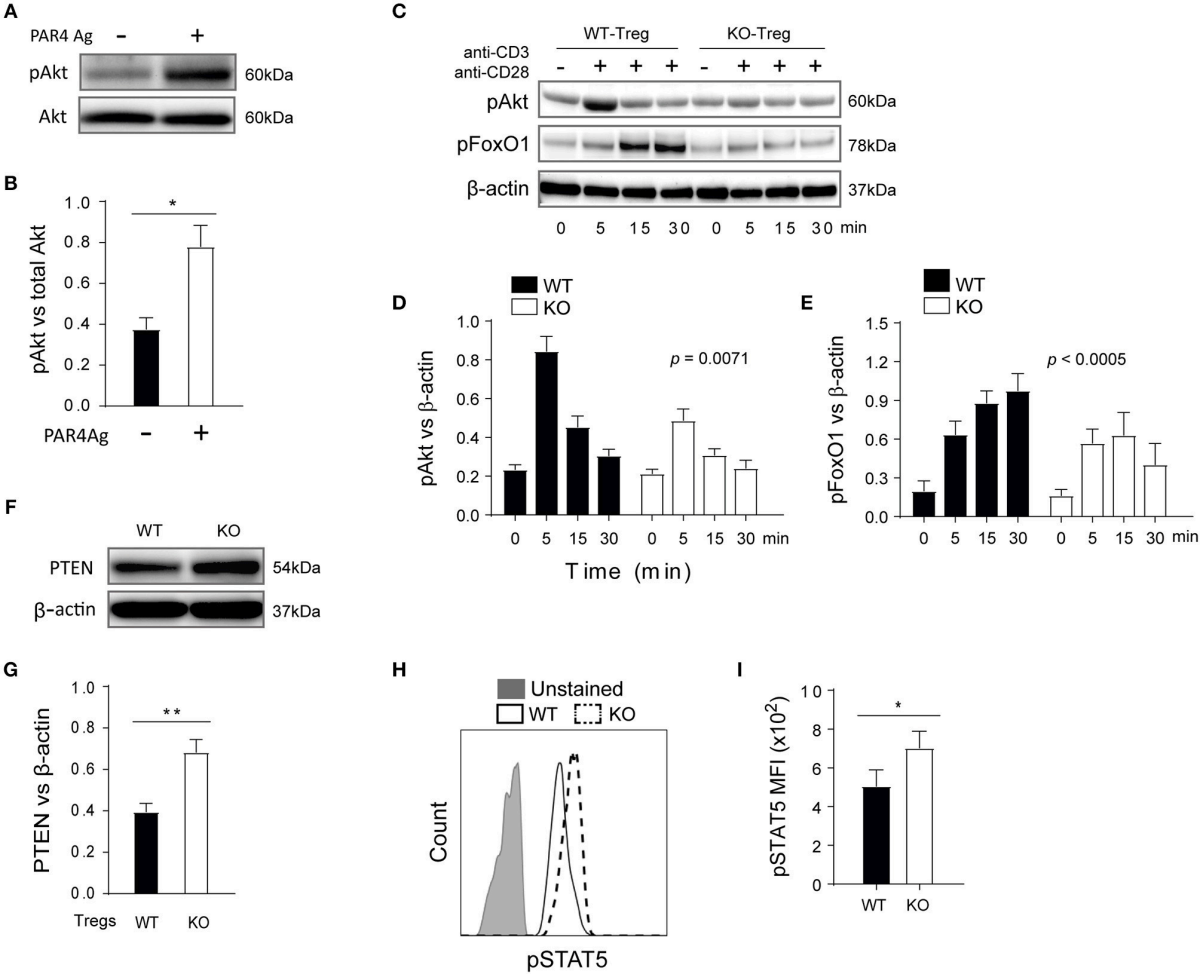
PTEN and Akt/FoxO1 links PAR4 signaling to regulate downstream pathways in WT Treg cells. Freshly isolated WT CD4^+^ CD25^+^ Tregs were stimulated with PAR4 agonist for 15 min. The levels of pAkt in these cells were analyzed by Western blots **(A)** and quantified against total Akt **(B)**. Graph shows mean ± SEM from four experiments. Data were analyzed by unpaired two-way *t*-test. ^*^p < 0.05 in comparison between WT and PAR4ko Tregs. The Tregs were also activated with anti-CD3/anti-CD28 for varied time points, and the levels of pAkt and pFoxo1 analyzed in western blots **(C)** and quantified against beta-actin [**(D,E)**, respectively]. Graphs show mean ± SEM from four experiments. Data were analyzed by two-way ANOVA. *P* = 0.0071 and *p* < 0.0005, compared between WT and PAR4ko Tregs. The level of total PTEN in the Treg cells from WT and PAR4ko mice was analyzed in Western blots **(F)** and quantified against beta-actin **(G)**. Graph shows mean ± SEM from four experiments. Data were analyzed by unpaired two-way *t*-test. ^**^p < 0.001 in comparison between WT and PAR4ko Tregs. The Tregs were also activated with IL-2 for 30 min and then the MFI levels of pSTAT5 were analyzed by flow cytometry **(H)** and the cumulative data **(I)**. Graph shows mean ± SEM from three experiments. Data were analyzed by unpaired two-way *t*-test. ^*^p < 0.05 in comparison between WT and PAR4ko Tregs.

Highly pure WT Tregs were stimulated with the PAR4 agonist AY-NH2 (50 μM) for 15 min and phosphorylation of Akt was measured by Western Blot (Figure 6A). Significantly higher levels of Akt phosphorylation were observed in the presence of the PAR4 agonist (*p* = 0.0267; Figure 6B), compared to controls, confirming that PAR4 engagement indeed triggered activation of the PI3K/Akt pathway. To further investigate the downstream consequences of this pathway, WT and PAR4ko Tregs were activated with plate-bound anti-CD3/CD28 antibodies and the phosphorylation status of Akt and FoxO1 was analyzed (Figure 6C). Significantly higher levels of pAkt and pFoxO1 were observed in WT Tregs compared to PAR4ko Tregs, with maximum phosphorylation at 5 and 30 min post-activation, respectively (*p* = 0.0071 and *p* < 0.0005; Figures 6D,E, respectively). Consistent with these findings, the levels of PTEN were higher in PAR4ko Tregs compared to WT Tregs (*p* = 0.0082; Figures 6F,G).

STAT5 phosphorylation is another intracellular signaling pathway that has been shown to directly regulate the expression and stability of FoxP3 through binding to the *FoxP3* promoter (51). To assess whether this pathway was affected by the absence of PAR4, the phosphorylation of STAT5 was measured in WT and PAR4ko Tregs following stimulation with 20 U/ml IL-2 for 30 min, as previous studies have shown that IL-2 signaling is required for Treg development and function (52, 53). PAR4ko Tregs had higher level of pSTAT5 after activation compared to WT Tregs (*p* = 0.0474, Figures 6H,I). Taken together, these observations show that PAR4 mediated PI3K/Akt signaling leads to inhibition of FoxO1, PTEN and STAT5 activities, resulting into the functional defect observed in PAR4^+^ Tregs.

## Discussion

Our study demonstrates for the first time the inhibitory role that signaling through PAR4 has on Treg function. PAR4-deficient mice were shown to have higher percentages of Tregs in the spleens, peripheral and mesenteric LNs and PAR4ko Tregs expressed higher levels of CD25, CD62L and CD73 molecules compared to WT Tregs. We demonstrated that PAR4ko Tregs had an enhanced suppressive capacity compared to WT Tregs *in vitro* and *in vivo*. These results obtained with the PAR4ko Tregs were confirmed by using WT Tregs and an antagonist of PAR4. Finally the enhanced function of Tregs in the absence of PAR4 signaling was associated with reduced PAR4-medited Akt phosphorylation and an increased activity of FoxO1 and PTEN. The results presented here highlight the importance of PAR4 ligation for the function of Tregs and possibly other cells of the adaptive immune system.

PAR4 was initially described as a secondary thrombin receptor which was less sensitive to thrombin-mediated activation than PAR1 (25). During blood coagulation, immune cells including Tregs can be exposed to a wide range of thrombin concentrations, from picomolar to micromolar levels of 0.8~1.4 μM (30, 54, 55). This vast range of thrombin concentrations can have very different effects on the function of local cells. Our data suggests that thrombin ligation of PAR4 can impair the function of Tregs in a similar manner to how platelets are affected by activation of PAR4 (55). Alternative serine proteases have also been reported to activate PAR4, such as Cathepsin G, which is released by neutrophils, and mannose-binding lectin-associated serine protease-1, an initiator of the complement lectin pathway (56, 57). Our data using peptide antagonists of PAR4 indicate that PAR4 is being activated in our experimental system. However, the lack of any effect of the highly specific thrombin inhibitor hirudin indicates thrombin is unlikely to be present in our culture system. In contrast, the capacity of the protease inhibitor cocktail to enhance Treg function suggests that an endogenous, as yet unidentified ligand of PAR4 is present in our experimental systems.

The increased immunoregulation and the presence of Tregs observed in murine transplant (39, 41) and EAE models (40) following high-dose ATIII or AAT treatment might be explained, at least in part, by the action of serine proteases influencing Treg function through signaling via PAR4. Furthermore, this suggests a physiological mechanism by which serine proteases, secreted systemically or locally, can impact Tregs or other immune cells. Indeed, we and others have shown that PAR signaling (PAR1 and 2) can modulate antigen uptake, priming, DC-mediated T cell activation and migration (43, 58, 59) further suggesting that PAR4 signaling may play an important role in immune balance.

The stability of FoxP3 and the high levels of expression of CD25 molecules are important elements in the maintenance of Treg function and protection from autoimmune disease (6, 7, 60, 61). The observation that these molecules are more stable or expressed at higher levels on WT Tregs treated with a PAR4 antagonist suggests that stimulation through PAR4 negatively regulates the stability and function of Tregs. This conclusion is further accentuated by the fact that PAR4-deficient Tregs express higher levels of functional molecules such as CD73 and CD62L, relative to WT Tregs.

The degradation of ATP, ADP and AMP to adenosine, achieved through the expression of CD73 and CD39 (62), and the subsequent interactions with the A2AR receptor, are important mechanisms by which Tregs function (63, 64). PAR4 signaling has been shown to inhibit adenosine signaling in a rat myocardial ischemia/reperfusion injury model (65), further supporting a negative effect of PAR4 signaling on Treg function.

CD62L expression is also important to determine suppressive capacity of Tregs. The CD62L^+^ subpopulation of Tregs has been shown to possess more potent suppressive and proliferative capacity *in vitro* than the CD62L^−^ (66) and only CD62L^+^ Tregs delayed the development of diabetes in an adoptive transfer model (67) and protected mice from acute GVHD (68). Our data showing that PAR4ko Tregs have higher levels of CD62L might imply a relationship between these previously documented observations and PAR4 signaling. More importantly, CD62L, together with CCR7, is a key homing receptor for Tregs to drive their migration to the secondary lymphoid tissues to suppress unwanted immune responses as well as acquire optimal immune regulatory activities (69–71). Higher expression of CD62L in PAR4ko Tregs, compared to WT Tregs, may provide an explanation for the increased number of FoxP3^+^ Tregs we detected in the peripheral lymphoid tissues of reconstituted Rag2^−/−^ mice.

**Figure 7:**
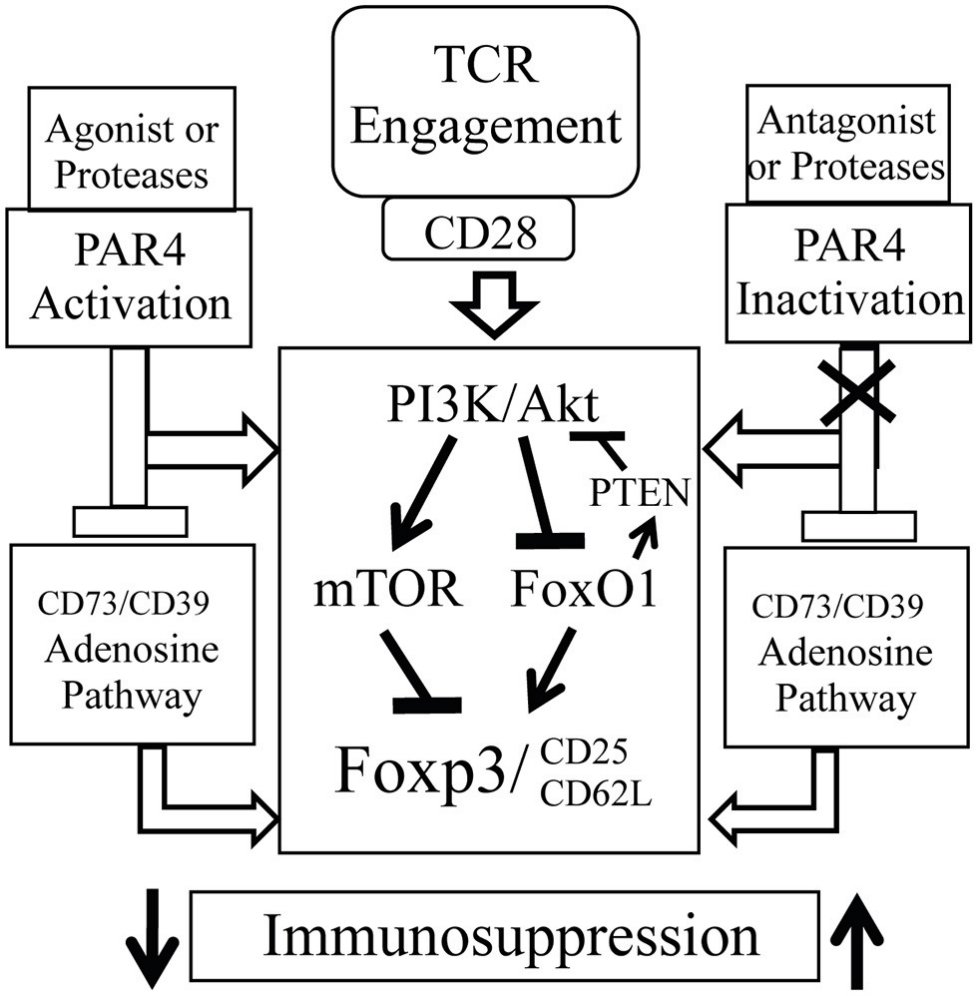
Possible underlying mechanism of PAR4 signaling. PAR4 activation may directly activate PI3K/AKT pathway, which then upregulates the activity of the downstream effector mTOR and downregulates the activities of Foxo1 and PTEN, and subsequently controls the expression of Foxp3, CD25, and CD62L. However, accumulation of excessive proteases at the sites of inflammation may overregulate PAR4 signaling on various cell types including Tregs, which then reduces function, stability, and mobility of Tregs. Moreover, PAR4 activation may also negatively control adenosine pathway. Once removing PAR4 signaling, the negative regulator, these Tregs possess “super” capacity with a Foxp3^high^ CD25^high^ CD62L^high^ phenotype and are able to induce immune tolerance.

Our findings also explored the possible mechanisms underlying the impact of PAR4 on Treg function. Although TCR/CD28-induced PI3K/Akt signaling is required for FoxP3 expression and Treg development, Akt activation is also known to inhibit FoxP3 expression by phosphorylation of the downstream transcription factor FoxO1 (20, 72) and mTOR1 (73, 74). The former is a positive regulator of FoxP3, the lipid phosphatase PTEN and the homing receptors CD62L and CCR7. We observed that stimulating WT Tregs with a PAR4 agonist induced phosphorylation of Akt which is in line with what has been observed in platelets (75, 76). Furthermore, PAR4-deficient Tregs had decreased levels of Akt activation, increased FoxO1 activity and an increased level of total PTEN compared to WT Tregs. Based on the evidence that FoxO1 is a pivotal regulator of Treg function (72) and from the results in our study, we propose that PAR4 signaling is a negative regulator of Treg function. Moreover, signaling via CD25 activates STAT5 to maintain FoxP3 expression in Tregs (51) and our data demonstrate that PAR4ko Tregs have increased level of pSTAT5, suggesting a mechanism by which PAR4ko Tregs have an enhanced stability of FoxP3 expression. In summary, we propose that PAR4-mediated Akt activation inhibits FoxO1 activity and consequently downregulates FoxP3, PTEN, CD25, and CD62L expression. Additionally, the interference with the adenosine and STAT5 pathways caused by PAR4 signaling is also likely to influence the function of Tregs (Figure 7).

Apart from TCR and IL-2 signaling pathways, PAR4 signaling is also likely that can cross-talk with several other signaling pathways. One possible candidate is TGF-beta as it plays a crucial role in the *in vitro* induction of Tregs (iTregs) (77, 78). However, *in vitro* TGFβ-induced FoxP3^+^ iTregs are lack of the epigenetic (79–81) and transcriptional (82, 83) signature of *in vivo* generated FoxP3^+^ Tregs, which is reflected by variable suppressive capacity (84) and unstable FoxP3 expression (79, 81, 85). Whether this feature is associated with PAR4 signaling needs further investigation.

In conclusion, our findings demonstrate a novel immunoregulatory role for PAR4 signaling in controlling the function of Tregs. It seems reasonable to speculate that PAR4-mediated signaling constrains the function of Tregs early in an inflammatory/immune response, thereby allowing an effective response to be generated against a pathogen. As inflammation subsides and PAR4 signaling diminishes, Tregs regain their potency and ability to prevent unwanted autoimmunity that might otherwise be triggered. Finally, the unique functional features exhibited by PAR4ko Tregs suggest that modulating PAR4 signaling in Tregs could have therapeutic benefit in autoimmunity and cancer.

## Ethics Statement

Animal work was undertaken in accordance to home office guidelines and under the home office license (PPL70/7302) which was also approved by the KCL ethics committee.

## Author Contributions

QP: conception and design, collection and assembly of data, data analysis and interpretation, and manuscript writing. KR: technical support. DB: data collection and analysis and intellectual input. JJ, ST, and DM: data collection and analysis. LS: data interpretation, intellectual input, and critical revision of the article. RL and AD: intellectual input and critical revision of the article. GL: conception and design and critical revision of the article for important intellectual contents.

### Conflict of Interest Statement

The authors declare that the research was conducted in the absence of any commercial or financial relationships that could be construed as a potential conflict of interest.
