# Supplementary material for: Protease Activated Receptor 4 as a Novel Modulator of Regulatory T Cell Function
Source: Front Immunol. 2019 Jun 18;10:1311. doi: 10.3389/fimmu.2019.01311 (PMC6591367; doi:10.3389/fimmu.2019.01311)
Supplement: Supplementary file 1 [file Data_Sheet_1.pdf]

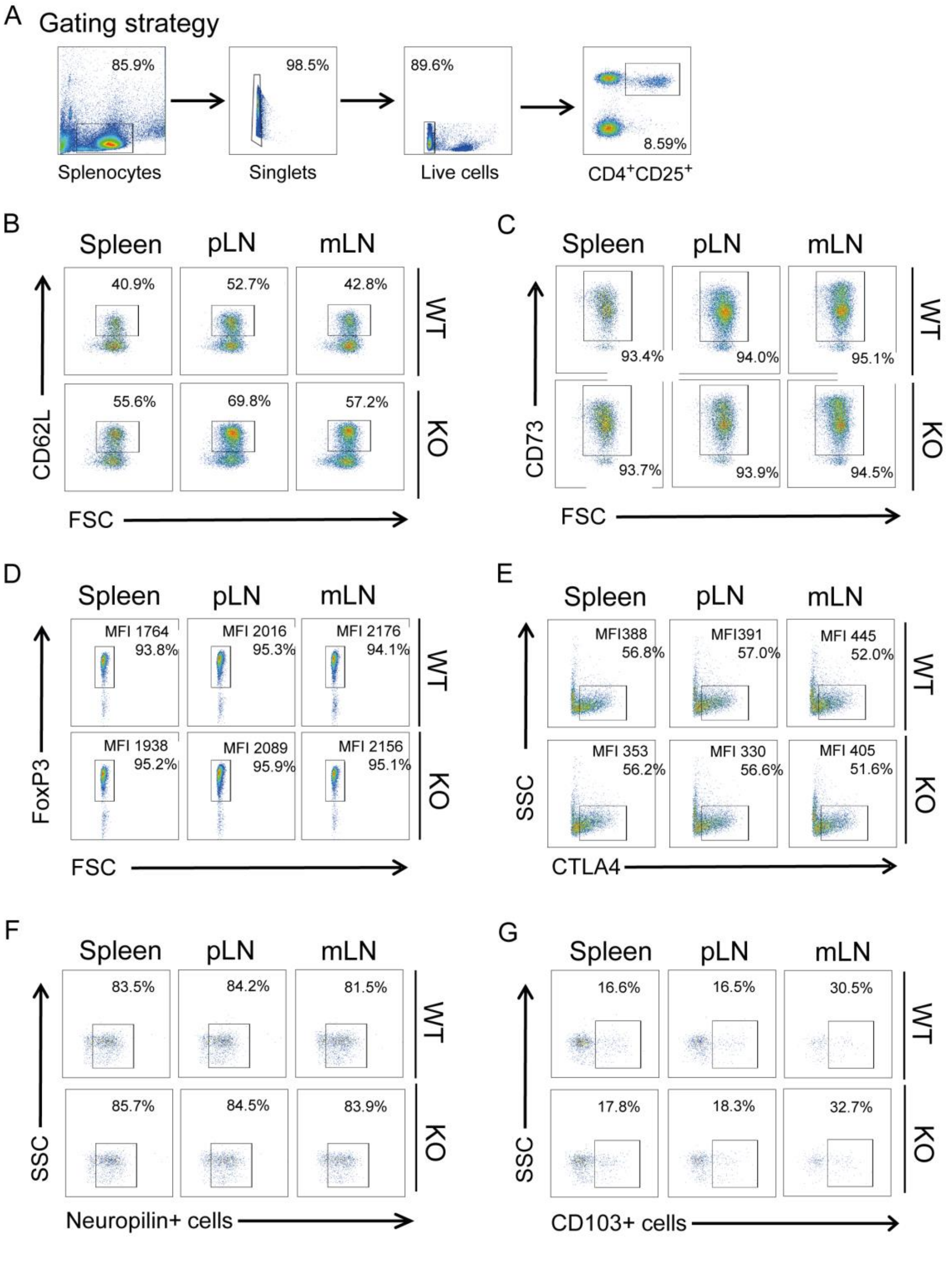

Suppl Figure 1. Characterization of cells Tregs in WT and PAR4ko mice. Gating strategy for $CD4^+CD25^+$ T cells (A). The percentage and MFI levels of functional markers FoxP3 (B), CD62L (C), CD73 (D), CTLA4 (E), Neuropilin (F) and CD103 (G) were further analysed in this population. Representative plots of the percentage and MFI levels of $FoxP3^+$ and $CTLA4^+$ subpopulations of $CD4^+CD25^+$ cells in spleen, pLN and mLN from WT and PAR4ko mice. Representative dot plots of the percentages of $CD62L^+$,$CD73^+$, $neuropilin^+$ and $CD103^+$ subpopulations of the $CD4^+CD25^+$ cells in spleen, pLN and mLN from WT and PAR4ko mice.

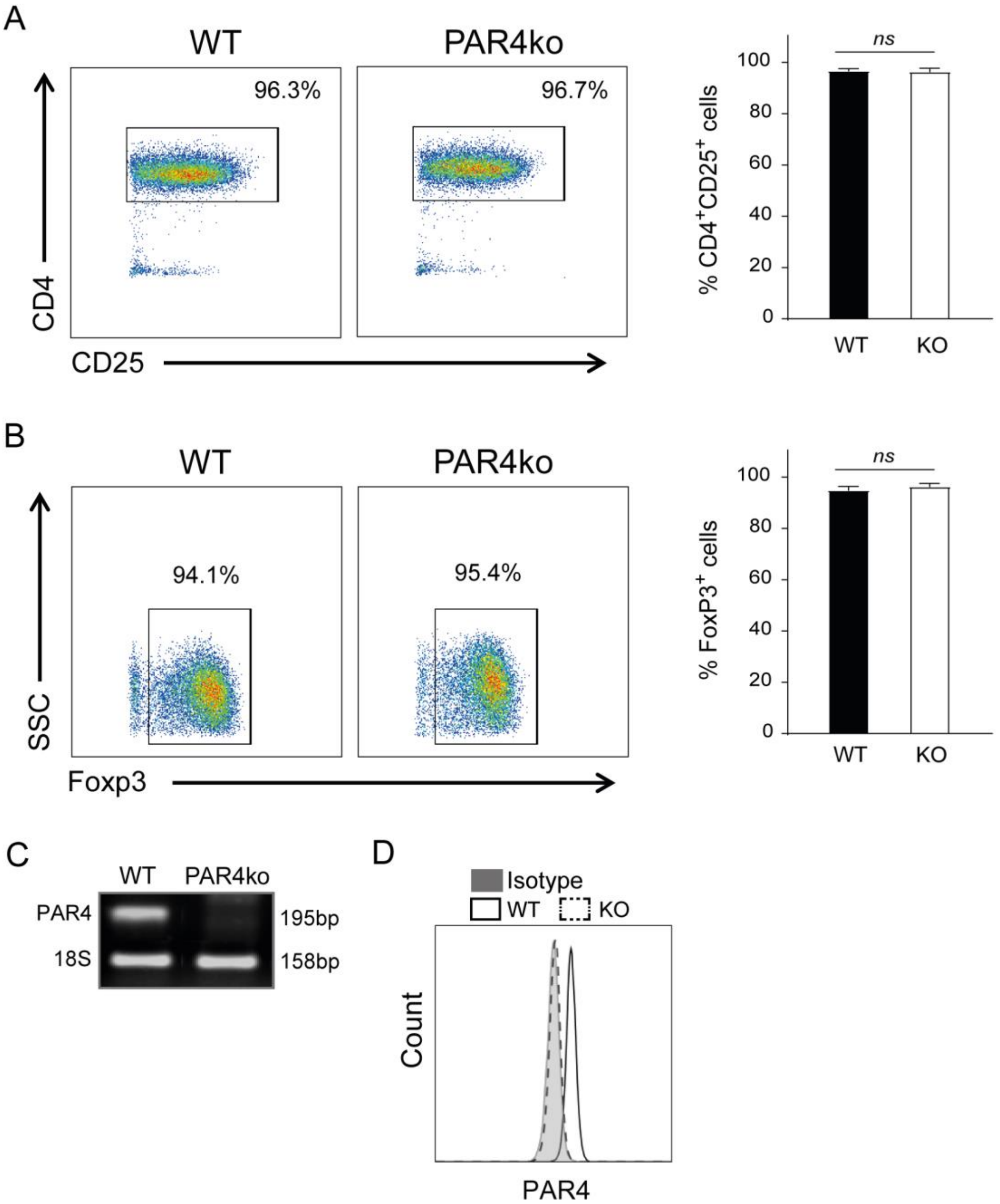

Suppl Figure 2. **Isolation, purity and expression of PAR4 of Tregs from WT and PAR4ko mice.** (A) Representative dot plots and pooled data of CD4$^+$CD25$^+$ cells isolated from spleen and pLN tissues of BL/6 WT or PAR4ko mice by sorting or using a murine CD4$^+$CD25$^+$ Treg cells kit. (B) Representative dot plots and pooled data of the percentage of FoxP3+ cells of freshly isolated CD4$^+$CD25$^+$ Treg. CD4$^+$CD25$^+$ Treg cells were freshly isolated from spleen and pLNs of WT and PAR4ko mice. Expression of PAR4 assessed at mRNA level (C) and protein level (D) in WT Tregs using PAR4ko Tregs as a negative control (dotted line) and unstained cells (grey filled histogram).

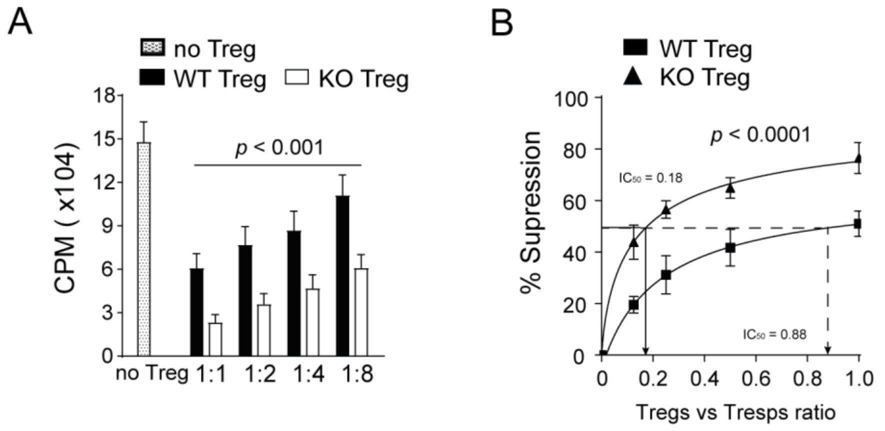

Suppl Figure 3. The enhanced suppressive capacity of PAR4ko Treg cells in tritiated thymidine uptake. $CD4^{+}CD25^{+}$ Tregs were purified from WT spleen and pLN and co-cultured with PAR4ko Tresps and APCs in the presence of anti-CD3 antibody. Representative histograms showing the count per minute (CPM) of thymidine uptake in Tresps cultured alone or in the presence of different numbers of WT or PAR4ko Tregs (A). Pooled data showing the percentages of suppression of WT and PAR4ko Tregs (B). IC50 values representing the Treg:Tresp ratio required to achieve 50% suppression are shown for the PAR4ko (triangles) and WT (squares) Tregs. Graphs show mean±SEM from six experiments. Data were analysed by two-way ANOVA, $p<0.001$ and $p<0.0001$, compared between these two Treg groups with a sequential dilution.

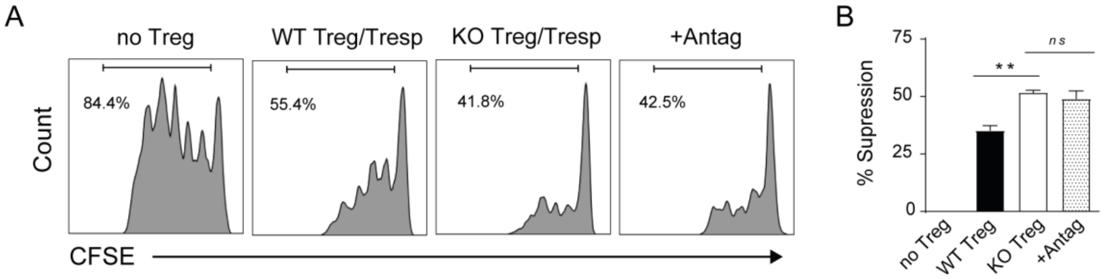

Suppl Figure 4. Specificity of PAR4 antagonist. $CD4^+CD25^+$ Tregs from WT and PAR4ko spleen and pLN were freshly isolated. Representative histograms (A) and pooled data (B) showing *in vitro* suppression of CFSE-labelled PAR4ko Tresps by WT (black or no filled bar; in the ratio of 2 to 1) or PAR4ko Tregs in absence or presence of a PAR4 antagonist (no filled or dot bar) in 72h suppressive assays co-cultured with PAR4ko APCs and addition of anti-CD3. Graphs represent mean±SEM from three experiments. Data were analysed by unpaired two-way *t* test. ** $p<0.005$ in comparison between the WT and PAR4ko Treg groups.
